# Immune-based cancer therapies: mechanistic insights, clinical progress, and future directions

**DOI:** 10.1186/s43046-025-00319-6

**Published:** 2025-09-29

**Authors:** Mahalakshmi Devaraji, Binoy Varghese Cheriyan

**Affiliations:** Saveetha College of Pharmacy, Saveetha Institute of Medical And Technical Sciences, Chennai, India

**Keywords:** Cancer immunotherapy, Immune checkpoint inhibitors, CAR-T cell therapy, Cancer vaccines, Cytokine therapies, Oncolytic viruses, Delivery modalities

## Abstract

The field of cancer immunotherapy has evolved rapidly, offering new treatment paradigms by harnessing the body’s own immune system to target and destroy malignancies. Various immunotherapeutic approaches, including immune checkpoint inhibitors, CAR-T cell therapy, cancer vaccines, cytokine therapies, and oncolytic viruses, have shown significant promise in treating different cancer types. This review provides a comprehensive examination of the historical development and recent advances in cancer immunotherapy. We discuss the mechanisms of action of key immunotherapeutic modalities, along with their clinical applications and innovative delivery techniques. In particular, we focus on immune checkpoint inhibitors, which have revolutionized the treatment of several cancers; CAR-T cell therapy, which has provided transformative results in hematological malignancies; and the potential of cancer vaccines, cytokine therapies, and oncolytic viruses. Additionally, the review addresses the current status of clinical trials and patents in the field, offering insight into the ongoing efforts to optimize these therapies for broader clinical use. Despite the promising results achieved, this review highlights significant challenges, such as immune-mediated toxicity, resistance to treatment, and the need for more effective delivery systems. While cancer immunotherapy has shown great potential in improving patient outcomes, overcoming existing obstacles such as toxicity and resistance remains a major challenge. This review offers a comprehensive overview of the state of cancer immunotherapy while also providing perspectives on its future directions and the ways in which these innovations may impact cancer treatment.

## Background

Cancer is a complicated and diverse illness that involves the uncontrolled proliferation of cells and the ability to invade or migrate to other areas of the body. It continues to be a major cause of illness and death globally [1, 2]. The treatment landscape for cancer is diverse and continually evolving, encompassing various modalities aimed at eradicating malignant cells while minimizing damage to healthy tissues. Cancer treatment strategies have historically included surgery, chemotherapy, and radiotherapy, but recent advancements in immunotherapy have reshaped the therapeutic landscape [3]. Immunotherapy, a way of treating cancer that uses the body’s immune system to fight it, has greatly changed the field of cancer care in the last few years [4, 5]. Unlike conventional therapies, immunotherapies primarily modulate the immune system’s ability to target and destroy cancer cells, although some, such as anti-programmed death-ligand 1 (PD-L1) inhibitors, directly target cancer cells. The natural ability of the immune system to identify and destroy cancer cells is instead enhanced or restored. This technique has demonstrated potential in the treatment of many forms of cancer and has several benefits, such as the possibility of achieving long-lasting remission and experiencing fewer adverse effects than traditional treatments do [6–8].

Immunotherapy encompasses several strategies, including immune checkpoint inhibitors, cancer vaccines, adoptive cell transfer, and cytokine therapies [9, 10]. Immune checkpoint inhibitors, such as pembrolizumab and nivolumab, inhibit proteins that prevent the immune system from fighting cancer cells. This makes immune reactions occur faster [11–13]. A person’s immune system is altered by adoptive cell transfer, making them more capable of identifying and eliminating cancer cells. Chimeric antigen receptor-T (CAR-T) cell treatment is an example that uses the above method [14]. The objective of cancer vaccines is to allow the immune system to react to certain cancer antigens. This helps the immune system find and fight cancer cells [15]. Chemicals such as interleukins and interferons are used in cytokine treatments to improve the ability of the immune system to fight cancer [16, 17].

The response of cancer patients to immunotherapy is crucial. This signifies a fundamental change in the field of oncology, providing optimism for patients who have tumors that do not respond to traditional therapies. Several forms of cancer, such as melanoma [18, 19], non-small cell lung cancer [20], and specific forms of leukemia and lymphoma [21], respond quite well to immunotherapy. Moreover, the development of combination therapies, where immunotherapy is used alongside other treatment modalities, is promising for enhancing treatment efficacy and overcoming resistance.

Despite its potential, immunotherapy has several challenges. The variability in patient responses, the development of resistance, and the potential for immune-related adverse effects are areas of active research and clinical concern [22–24]. However, the continuous progress in comprehending the processes of immune modulation and tumor immunology is creating opportunities for the development of more efficient and tailored immunotherapeutic approaches [25].

In addition, novel immunotherapeutic approaches are emerging. Personalized cancer vaccines, which are designed on the basis of the unique mutations present in an individual’s tumor, are being developed to improve the specificity and effectiveness of immune responses [26]. Bispecific antibodies, which can bind simultaneously to cancer cells and immune cells, are another promising innovation. These advancements aim to increase the precision and potency of immunotherapy, making it more effective for a broader range of patients [27].

This review aims to provide a comprehensive understanding of the role that immunotherapy has played in revolutionizing cancer treatment and to explore its potential to drive future advancements in oncology.

## Types of cancer immune theraphy

### Immune checkpoint inhibitors

Immune checkpoints are crucial regulatory pathways in the immune system that help maintain self-tolerance and prevent autoimmunity [28]. These pathways play crucial roles in controlling the duration and strength of immune responses, ensuring that the immune response can effectively respond to infections without causing substantial damage to the body's tissues. Cancer cells use different checkpoints in the body to evade detection and destruction by the immune system [29].

#### Mechanisms of action of immune checkpoint inhibitors

##### CTLA-4 pathway

One of the immunological checkpoint proteins that is produced on the surface of T cells is CTLA-4, which stands for cytotoxic T-lymphocyte antigen 4. It plays a crucial role in the process of suppressing immunological responses [30]. CTLA-4 competes with CD28, a stimulatory receptor on T cells, for binding to B7 molecules (CD80/CD86) on antigen-presenting cells (APCs). While CD28 binding to B7 molecules promotes T-cell activation and proliferation, CTLA-4 binding inhibits these processes. This inhibition is crucial for maintaining immune homeostasis and preventing autoimmunity [31]. However, in the context of cancer, the inhibitory function of CTLA-4 can be exploited by tumor cells to suppress antitumor immune responses, allowing them to grow and spread unchecked.

Anti-CTLA-4 antibodies, such as ipilimumab, block the interaction between CTLA-4 and B7 molecules [32]. These antibodies hinder the binding of CTLA-4 to B7, resulting in a decrease in the inhibitory signal and an increase in the stimulatory signal mediated by CD28. Consequently, there is an increase in the activation, reproduction, and endurance of T cells, enhancing the immunological reaction against malignant cells. Increased T-cell activity leads to the infiltration of tumors by stimulated T cells, hence facilitating the destruction of cancer cells [33, 34]. 

 Another important immunological checkpoint protein produced on the surface of T cells is called PD-1, which stands for "Programmed Death-1". Reduced T-cell activity results from an inhibitory signal transmitted when PD-1 binds to its agonists, PD-L1 and PD-L2. These ligands can be produced on cancerous cells or APCs [35, 36]. This interaction results in a reduction in the generation of cytokines and a decrease in the ability of T cells to multiply and survive. By exploiting the PD-1 pathway, cancer cells can effectively "turn off" T cells, escaping immune surveillance and destruction. The PD-1 pathway is thus a major mechanism by which tumors create an immunosuppressive microenvironment [37]. 

##### PD-1 pathway 

**Fig. 1:**
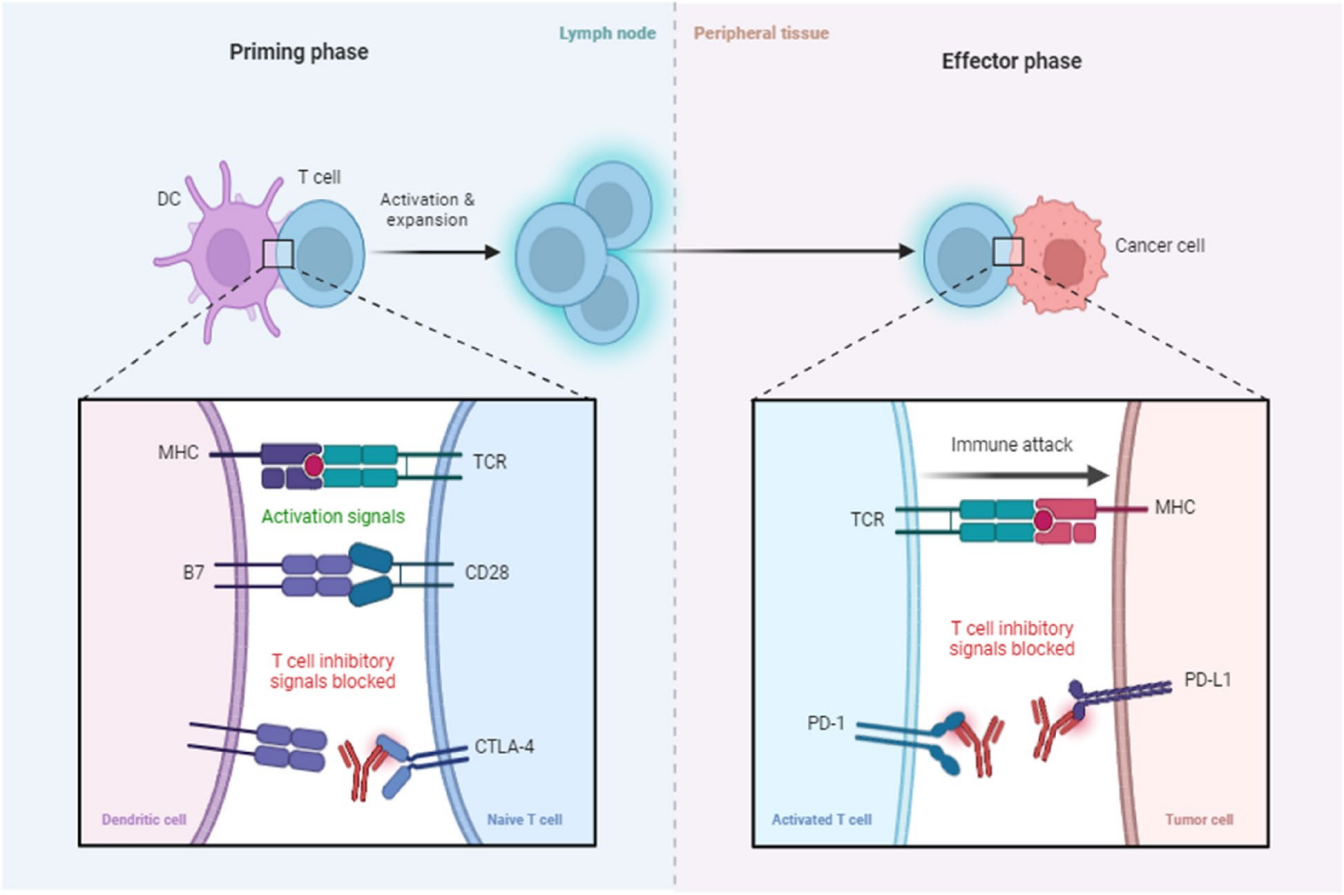
Mechanism of action of immune checkpoint inhibitors in cancer immunotherapy

Anti-PD-1 antibodies (e.g., pembrolizumab and nivolumab) and anti-PD-L1 antibodies (e.g., atezolizumab) block the interaction between PD-1 and its ligands [38]. These antibodies restore T-cell activation and function by blocking this pathway, which in turn decreases inhibitory signals. This reactivation of T cells leads to enhanced recognition and destruction of tumor cells. Inhibiting the interaction between PD-1 and PD-L1 also leads to increased cytokine production, hence enhancing the fight of the immune system against cancer [39, 40]. The mechanisms described are illustrated in Figure 1, which shows the phases of T-cell activation and the immune response in cancer immunotherapy.

#### Newer immune checkpoints: TIM-3, LAG-3, and TIGIT

The landscape of cancer immunotherapy has expanded significantly with the identification of newer immune checkpoints, including T-cell immunoglobulin and mucin-domain containing-3 (TIM-3), lymphocyte-activation gene 3 (LAG-3), and T-cell immunoreceptor with Ig and ITIM domains (TIGIT). These checkpoints provide critical insights into the complex mechanisms of immune regulation and resistance within the tumor microenvironment, representing promising therapeutic targets in the ongoing fight against cancer [41]. 

##### LAG-3

LAG-3 is another critical checkpoint that negatively regulates T-cell activation and proliferation. It is expressed on activated T cells, regulatory T cells (Tregs), and some antigen-presenting cells. LAG-3 binds to MHC class II molecules with a higher affinity than does CD4, effectively competing for binding and inhibiting T-cell activation [42–44]. Its role in T-cell exhaustion and immune suppression in the tumor microenvironment is well documented, as LAG-3 expression often correlates with poor clinical outcomes in various malignancies [45–47]. LAG-3 inhibitors are being developed as monotherapies and in combination with PD-1 inhibitors [48, 49]. Early clinical trials have shown promising results, indicating that LAG-3 blockade can enhance T-cell function and improve responses to immunotherapy in cancers such as melanoma and non-small cell lung cancer. Researchers are also exploring the potential of combining LAG-3 inhibitors with other immunotherapeutic agents to overcome resistance mechanisms and improve overall survival rates [50–52].

##### TIGIT

TIGIT is a coinhibitory receptor expressed on T cells and natural killer (NK) cells that functions to dampen immune responses by inhibiting T-cell activation and promoting immune tolerance [53]. It competes with CD226 (also known as DNAM-1) for binding to shared ligands, such as PVR (poliovirus receptor) and Nectin-2, which are upregulated in many tumors [54]. The engagement of TIGIT results in the inhibition of cytotoxic T-cell activity and promotes the expansion of regulatory T cells, thereby fostering an immunosuppressive TME. Targeting TIGIT with monoclonal antibodies is an area of active research, with several trials underway evaluating its efficacy in various cancers, including hematologic malignancies and solid tumors [55]. Early studies suggested that combining TIGIT inhibitors with PD-1/PD-L1 inhibitors or other immunotherapeutic agents may enhance antitumor immunity and help overcome resistance to existing therapies. As clinical data emerge, understanding the optimal patient populations and treatment combinations will be critical for maximizing the benefits of TIGIT blockade in cancer therapy [56].

### CAR-T-cell therapy

CAR-T (chimeric antigen receptor (CAR-T) cell) therapy is a groundbreaking form of adoptive cell transfer that significantly enhances the ability of a patient’s T cells to fight cancer [57]. This innovative therapy involves several intricate steps, starting with the collection of T cells from the patient’s blood. Once harvested, these T cells are genetically modified in a laboratory to express chimeric antigen receptors (CARs) on their surface. The unique capacity of T cells to identify and target cancer cells can be achieved through the use of CARs, which are synthetic receptors [58, 59]. For T cells to attach to a particular antigen in cancer cells, the CAR structure incorporates an antigen-binding domain, which is usually obtained from a monoclonal antibody. Next, there is a hinge region that provides flexibility, a transmembrane domain that ensures a binding location in the cell's membrane, and intracellular signaling regions that play a vital role in T-cell activation and proliferation, such as CD3ζ and costimulatory domains such as CD28 or 4-1BB [60, 61].

**Fig. 2:**
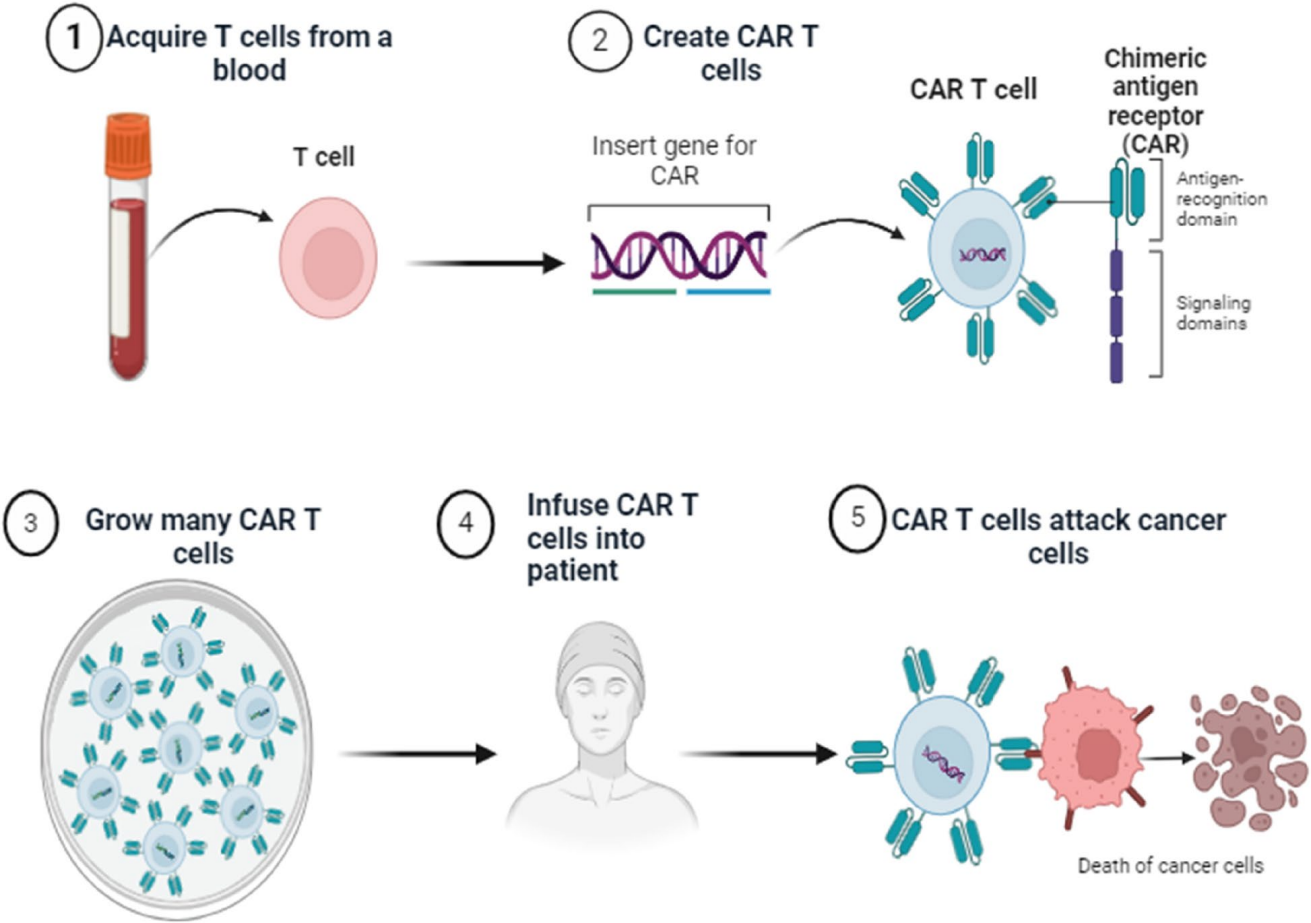
This figure outlines the process of CAR-T-cell therapy, a personalized cancer treatment. The procedure involves five main steps: (1) acquiring T cells from the patient's blood, (2) genetically modifying the T cells to express chimeric antigen receptors (CARs), (3) expanding the number of CAR-T cells, (4) infusing the CAR-T cells back into the patient, and (5) allowing the CAR-T cells to recognize and attack cancer cells, leading to destruction of the tumor.

Following their modification, CAR-T cells are expanded in the laboratory before being reintroduced to patients. Cancer antigen-recognition T cells (CAR-T cells) bind to cancer cells expressing their target antigen, are activated, and then secrete cytotoxic substances that kill the cancer cells. The highly focused strategy has demonstrated exceptional efficacy in managing specific blood cancers, offering renewed optimism for individuals with previously resistant tumors [62]. This process is visually represented in Figure 2, highlighting the step-by-step procedure of CAR-T-cell therapy.

#### CAR construction

 The CAR-T-cell therapy technique involves the extraction of T cells from the patient's bloodstream. These T cells subsequently undergo genetic modification under laboratory conditions to enable the expression of chimeric antigen receptors (CARs) on their cell surface. CARs are artificial receptors that give T lymphocytes the unique ability to selectively recognize and attack particular proteins found in cancer cells [63]. The construction of a CAR is a meticulous process, ensuring that each component functions synergistically to increase the efficacy of T cells against cancer. The initial component of a CAR's antigen-binding domain is its initial component. A monoclonal antibody is the usual source for this domain, which enables it to identify and attach to a particular antigen found on cancer cell surfaces. Precise binding specificity is essential for guiding CAR-T cells toward their intended targets and ensuring that they exclusively attack cancer cells, hence minimizing adverse effects on healthy organs. Following the antigen-binding domain is the hinge region, a flexible linker that provides the necessary mobility for the CAR to properly orient itself when engaging with the antigen. This flexibility is essential for the effective binding and subsequent activation of T cells [64]. 

#### Mechanism of action

Once equipped with CARs, T cells undergo a transformative process in CAR-T-cell therapy, aiming to combat cancer with precision and potency. Following genetic modification in the laboratory to express CARs, these T cells are cultivated to reach sufficient numbers for therapeutic effectiveness [65, 66]. Moreover, patients typically undergo a preparative regimen, often involving chemotherapy, which serves to reduce the existing population of lymphocytes and create space for incoming CAR-T cells upon reintroduction into the bloodstream [67]. The therapeutic mechanism of CAR-T cells is executed via a sequence of carefully coordinated procedures. After being reintroduced into the body, CAR-T cells travel through the bloodstream and aggressively search for cancer cells that have a particular antigen targeted by the CARs. The antigen-binding domain of the chimeric antigen receptor (CAR) plays a crucial role in this stage, allowing CAR-T cells to identify and attach to the antigen on the outer layer of cancer cells with exceptional precision. Upon binding to their specific antigen, CAR-T cells activate an internal signaling cascade via their CD3ζ and costimulatory regions (such as CD28 or 4-1BB), which leads to their activation. This activation triggers rapid and extensive multiplication of CAR-T cells, resulting in a significant increase in their quantity. This increase helps generate a strong and effective immune system defense against malignancy. The cytotoxic process is a crucial component of CAR-T-cell therapy. Upon encountering cancer cells, activated CAR-T cells produce cytotoxic granules that contain perforin and granzymes. These molecules can penetrate cancer cells, inducing apoptosis or programmed cell death. Furthermore, CAR-T cells secrete cytokines such as interferon-gamma and interleukins, which enhance the overall efficacy of therapy by amplifying the immune response and recruiting additional immune cells to the tumor location. The sustained immune response generated by CAR-T cells ensures continued surveillance and destruction of cancer cells bearing the targeted antigen. This prolonged activity offers a potential advantage over conventional therapies by potentially achieving durable remission in patients with otherwise treatment-resistant cancers. Ongoing research continues to refine CAR-T-cell therapy and explore improvements in efficacy, safety, and applicability across various cancer types, promising further advancements in personalized cancer treatment strategies.

#### Evolution of CAR-T-cell development

**Fig. 3:**
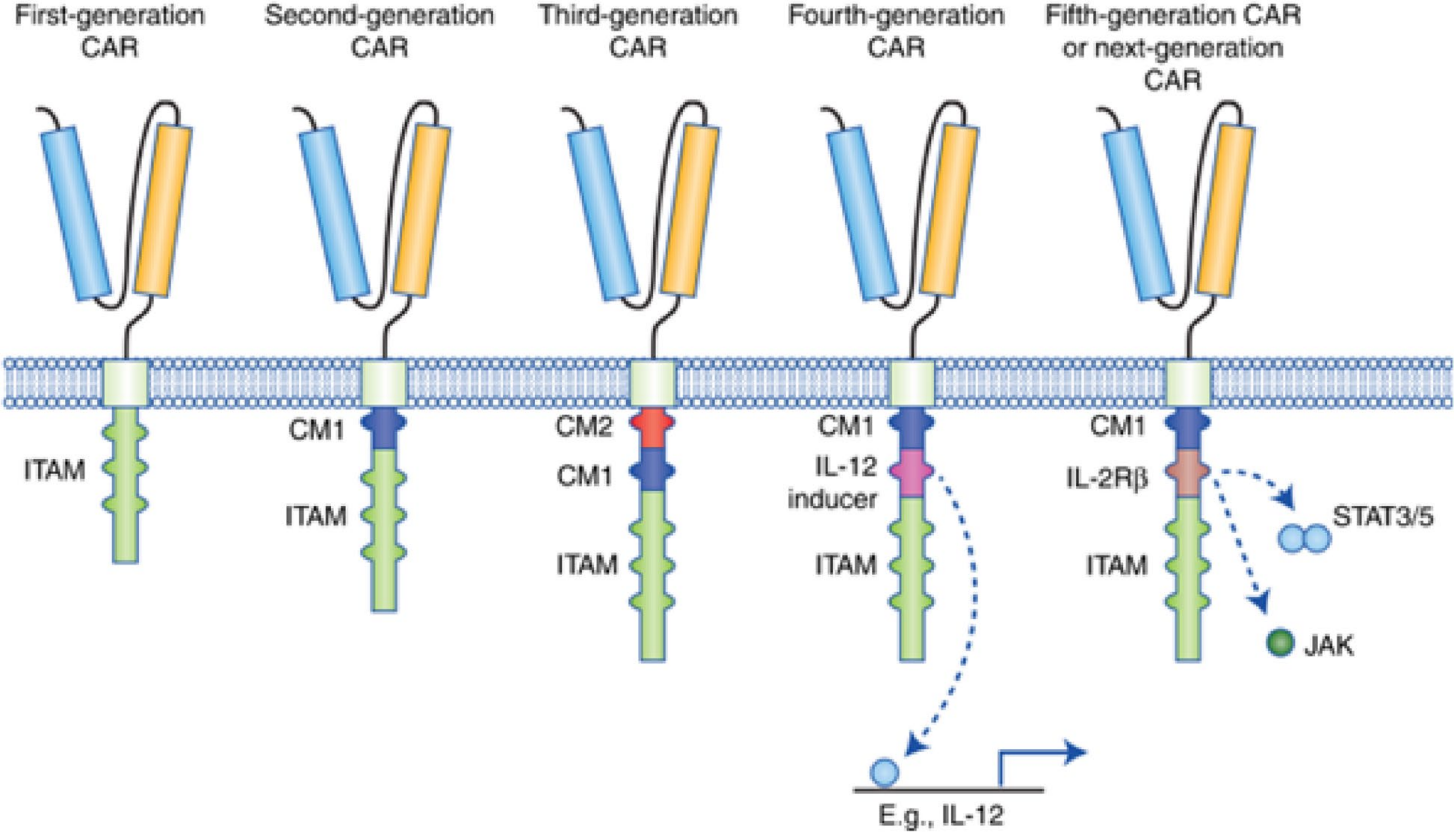
Structures of different chimeric antigen receptor (CAR) generations. [74]

The advancements in CAR development over the last thirty years can be broadly categorized into five generations on the basis of the structure and composition of the endodomain. The initial generation of CARs comprises a solitary CD3ζ intracellular domain [68]. Preliminary investigations with first-generation CAR-T cells demonstrated diminished cytotoxicity and proliferation due to insufficient costimulatory (e.g., CD27, CD28, CD134, and CD137) and cytokine (e.g., interleukin (IL)-2) signaling [68, 69]. A second generation of CARs was developed to improve T-cell proliferation and cytotoxicity by incorporating a costimulatory domain, such as portions of CD28 or CD137, into the intracellular signaling domain [70–72]. The third generation of CARs enhanced the second generation by including a third intracellular signaling sequence with an extra costimulatory domain, such as CD134 or CD137 [68]. The fourth generation of CARs is derived from second-generation CARs and incorporates a protein, such as interleukin 12 (IL-12), which is expressed either constitutively or inducibly following CAR activation. T cells modified with these fourth-generation CARs are designated T cells redirected for universal cytokine-mediated killing (TRUCKs). The activation of these CARs enhances the production and release of target cytokines to facilitate tumor eradication through many synergistic processes, including exocytosis (perforin, granzyme) and death ligand–death receptor (Fas–FasL, TRAIL) pathways [73]. TRUCKs will be addressed in a subsequent section. A fifth generation of CARs is presently under investigation; these are derived from the second generation of CARs, which incorporate a shortened cytoplasmic IL-2 receptor β-chain domain with a binding site for the transcription factor STAT3. The antigen-specific activation of this receptor concurrently initiates TCR signaling (via the CD3ζ domains), costimulatory signaling (CD28 domain), and cytokine signaling (JAK–STAT3/5), thereby delivering the three synergistic signals necessary for complete T-cell activation and proliferation [72]. Further versions of the previously mentioned CARs, including dual CARs, split CARs, and inducible-split CARs, have been developed to augment the specificity and regulation of the transfused T cells. The CARs will be elaborated upon in further depth below. Figure 3 shows the structures of different chimeric antigen receptor (CAR) generations.

Over the past thirty years, CARs have evolved from their initial characterization to receiving FDA approval for patient usage. Despite these advancements, the innovative CAR designs and enhancements shown in recent generations, as demonstrated in vitro or in animal models, have not been validated in human patients. No study has yet conducted a clinical comparison of first- or subsequent-generation CARs targeting a single antigen, hindering an accurate assessment of the various CAR generations and complicating the selection of appropriate combinations for future clinical studies. Consequently, the evaluation of a specific CAR generation relies on preclinical animal models rather than clinical evidence. At present, it is uncertain which design yields the optimal clinical benefit for patient outcomes.

#### Adoptive Cell Therapy in Cancer Immunotherapy

Adoptive cell therapy (ACT) is a cutting-edge approach in cancer immunotherapy in which immune cells are extracted, modified, and reinfused into patients to target tumors. The key types of ACT include CAR-T, TCR-T, CAR-NK, and CAR-M therapies.[75]

#### TCR-T (T-cell receptor-engineered T cell) therapy

TCR-T-cell therapy is another promising form of adoptive cell therapy that differs from CAR-T-cell therapy in that it focuses on the natural mechanism of T-cell recognition. TCR-T cells are engineered to express T-cell receptors (TCRs) that can recognize tumor-specific antigens presented by patients’ MHC molecules. This allows TCR-T cells to target a broader range of tumor antigens, including intracellular proteins, which are inaccessible to CAR-T cells [76]. TCR-T-cell therapy is particularly useful for targeting tumors that express specific shared antigens, such as NY-ESO-1 or MART-1, which are commonly found in cancers such as melanoma and sarcoma [77]. However, the dependency on MHC means that TCR-T cells are limited by the patient's HLA type, and variations in antigen presentation can affect treatment efficacy. Additionally, tumors can evolve to downregulate MHC expression as an immune evasion mechanism, limiting the long-term effectiveness of TCR-T-cell therapy. Despite these challenges, TCR-T-cell therapy holds great promise, particularly in the treatment of solid tumors where CAR-T cells have struggled [78, 79].

#### **CAR-NK (chimeric antigen receptor natural killer) cell therapy**

CAR-NK therapy is an emerging form of adoptive cell therapy that leverages the innate immune capabilities of natural killer (NK) cells. Like CAR-T cells, CAR-NK cells are engineered to express a CAR that allows them to specifically target tumor antigens [80]. However, NK cells have the unique ability to kill tumor cells without prior sensitization and do not rely on MHC recognition, making them more versatile in targeting a wide range of cancers. Additionally, CAR-NK cells have a lower risk of causing severe side effects, such as cytokine release syndrome (CRS) and graft-versus-host disease (GVHD), which are common concerns with CAR-T-cell therapy [81]. This reduced risk makes CAR-NK cells potentially safer, and they can be derived from both autologous (patient-derived) and allogeneic (donor-derived) sources, suggesting the possibility of "off-the-shelf" treatments. Early clinical trials have shown encouraging results in treating B-cell malignancies, particularly in targeting CD19-positive cancers, and ongoing research is exploring their application in solid tumors as well. CAR-NK therapy represents a promising alternative or complement to CAR-T-cell therapy, particularly for patients who may not be candidates for T-cell-based therapies [82].

##### **CAR-M (chimeric antigen receptor macrophage) therapy**

CAR-M therapy is a novel and still-developed form of adoptive cell therapy that modifies macrophages to recognize and attack cancer cells. Macrophages, as part of the innate immune system, play a critical role in phagocytosis (engulfing and digesting pathogens or dead cells) and can also present tumor antigens to other immune cells, promoting a broader immune response [83]. An issue with CAR-macrophage-based therapy is that macrophages can phagocytize only portions of targeted cells. To mitigate this constraint, the combination of an anti-CD47 antibody and CAR-T cells was used to enhance the phagocytosis of whole cells [84]. Another technique involves the engulfment of receptor intracellular domains into the CAR construct to initiate the engulfment of whole cells. For example, a CAR incorporating Megf10 and the ɣ component of Fc receptors (chimeric antigen receptor-phagocytes, CAR-Ps) was designed for macrophages to initiate phagocytosis of cells expressing particular antigens. Additionally, CAR-Ps are associated with the PI3K p85 subunit to create a "tandem" CAR (CAR-Ptandem) capable of efficiently engulfing human cancer cells [85].

### Cancer vaccines

As a state-of-the-art strategy in oncology, cancer vaccines target cancer cells by directing the body's immune system to destroy them. These vaccines are designed to stimulate the immune system to fight against antigens that are specific to cancer cells. They have the potential to be used for both preventing and treating cancer [86].

#### Types of cancer vaccines

Cancer vaccines can be classified into two main categories: preventative and therapeutic. Every iteration of treatment seeks to use the immune system's capacity to identify and eradicate cancerous cells; however, their methodologies and desired results vary [87].

#### Preventive vaccines

Preventive cancer vaccines are designed to prevent cancer development by targeting specific viruses or pathogens known to cause cancer. The most notable example of a preventive cancer vaccine is the human papillomavirus (HPV) vaccine. HPV is a common virus that can lead to cervical cancer, as well as other cancers, such as anal, vaginal, vulvar, penile, and oropharyngeal cancers [88, 89]. HPV vaccination functions by stimulating the immune system to fight against the virus, preventing HPV infection and the consequent formation of malignancies linked to HPV. Other examples of preventive cancer vaccines may target viruses such as hepatitis B virus (HBV) and hepatitis C virus (HCV), which can lead to liver cancer (hepatocellular carcinoma) [90]. These vaccinations successfully lower the chance of developing specific types of cancer by avoiding viral infections that are associated with cancer development. This strategy for cancer prevention is proactive. Three FDA-approved vaccines are available to protect against HPV infection: Gardasil 9 and Cervarix.Gardasil was the first HPV vaccine approved in 2006 [91].

#### Therapeutic vaccines

**Fig. 4:**
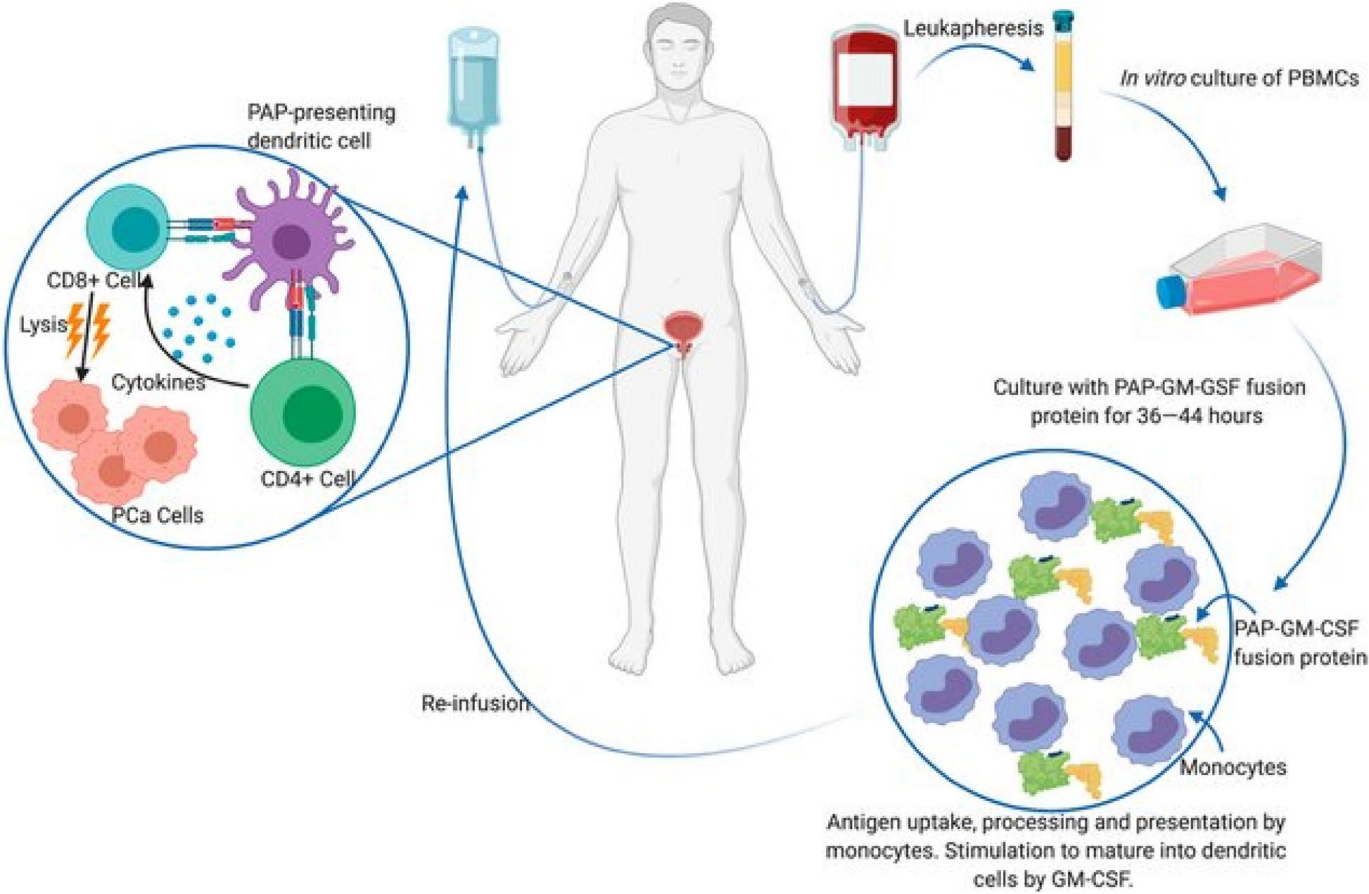
Dendritic cell-based immunotherapy for prostate cancer

In contrast, therapeutic cancer vaccines are designed to treat existing cancers by stimulating the immune system to recognize and attack cancer cells that express specific antigens. These vaccines aim to enhance the body's natural immune response against cancer cells, potentially slowing disease progression or inducing remission. A therapeutic cancer vaccine functions by stimulating the immune system to target cancer cells through the introduction of specific antigens exclusive to tumors [92]. These antigens can originate from proteins or other substances that are produced only by cancer cells. Therapeutic vaccines commonly incorporate adjuvants, which are chemicals that stimulate and augment the immunological response to the antigen, to increase immune activity. Dendritic cells and other antigen-presenting cells (APCs) are known to play important roles in the development of therapeutic vaccines [93]. They collect and analyze antigens that are specific to cancer and then present these antigens to T cells in a manner that stimulates them to identify and destroy cancerous cells throughout the entire body. Initiating a targeted and long-lasting immune response against cancer is the goal of this therapy, which has the potential to reduce or eradicate the tumors shown in Figure 4. One notable example of a therapeutic cancer vaccine is Sipuleucel-T (Provenge), which is approved for the treatment of metastatic castration-resistant prostate cancer. Sipuleucel-T is a procedure in which a patient's dendritic cells are collected and then exposed to a prostate cancer-specific antigen (PAP) that is combined with an immune-stimulating agent called granulocyte‒macrophage colony‒stimulating factor (GM-CSF). After being activated, these dendritic cells, which are loaded with antigens, are reintroduced into the patient's body. They then trigger a specific immune response against prostate cancer cells that express PAP [94, 95].

Various types of therapeutic vaccines are being developed, including peptide vaccines, cell-based vaccines, mRNA vaccines, and DNA vaccines. Each of these approaches has unique mechanisms and advantages, as well as challenges that researchers are actively addressing.

#### Peptide vaccines

Peptide vaccines consist of short sequences of amino acids (peptides) that correspond to specific tumor-associated antigens (TAAs) or neoantigens. By presenting these peptides to the immune system, the vaccine aims to elicit a targeted immune response against cancer cells expressing those antigens. Peptide vaccines can be customized on the basis of the individual patient’s tumor profile, which enhances their specificity and potential effectiveness. A notable example is the peptide vaccine targeting prostate-specific antigen (PSA), which is used in patients with prostate cancer. While peptide vaccines have shown promise in clinical trials, challenges remain in achieving robust and durable immune responses, as well as in identifying suitable antigens for different cancer types [96].

##### **Cell-based vaccines**

Cell-based vaccines, including dendritic cell vaccines and whole tumor cell vaccines, leverage the ability of immune cells to activate and educate the immune system against cancer. Dendritic cell vaccines involve isolating dendritic cells from patients, loading them with tumor antigens, and reinfusing them to stimulate T-cell activation [97]. Whole tumor cell vaccines are derived from a patient’s own tumor cells or genetically modified tumor cells that express antigens to provoke an immune response. These approaches have the advantage of presenting a broad array of antigens to the immune system, potentially overcoming the limitations of single-antigen targeting [98]

##### **mRNA vaccines**

mRNA vaccines represent a cutting-edge approach to cancer immunotherapy. These vaccines use messenger RNA (mRNA) to instruct cells to produce specific tumor antigens, thereby eliciting an immune response. The mRNA can be delivered in lipid nanoparticles, which facilitate their cellular uptake. Notably, mRNA vaccines have gained significant attention because of their rapid development during the COVID-19 pandemic and are now being explored in oncology [99]. Personalized mRNA vaccines can be designed on the basis of individual tumor sequencing, allowing for the targeting of patient-specific neoantigens. Early clinical trials have shown promise in various cancers, including melanoma and non-small cell lung cancer, but further research is needed to optimize their efficacy and assess long-term responses [100, 101].

##### **DNA vaccines**

DNA vaccines involve the direct introduction of plasmid DNA encoding tumor-associated antigens into the body. Once inside, the host cells use DNA to produce the corresponding antigens, triggering an immune response against the tumor. This approach is appealing because of its stability and ease of production. DNA vaccines can be designed to target specific cancer antigens and may be delivered via intramuscular injection or electroporation to increase uptake. While DNA vaccines have shown potential in preclinical studies and early-phase clinical trials, challenges remain in ensuring sufficient immune activation and overcoming potential tolerance mechanisms [102, 103].

### Cytokine Therapies in Cancer Treatment

Cytokine therapies employ signaling proteins called cytokines, which have essential functions in controlling immune responses, to increase the body's natural defenses against cancer. Interleukins (ILs) and interferons (IFNs) are crucial cytokines employed in cancer therapy [104]. These cytokines possess unique modes of action and are utilized to treat different types of tumors.

Interleukins (ILs) and interferons (IFNs) are pivotal cytokines employed in cancer therapy because of their potent immunomodulatory properties. IL-2 and IFN-α are among the most extensively studied and utilized cytokines in oncology [105]. Interleukin-2 (IL-2) plays a critical role in promoting the proliferation and activation of T cells and natural killer (NK) cells. By binding to IL-2 receptors on these immune cells, IL-2 enhances their cytotoxic activity against cancer cells. Owing to the ability of this cytokine to increase the number of effector T cells, which can selectively recognize and target cancer cells, the immunological response of the body to tumors is strengthened [106, 107]. Interferon-alpha (IFN-α) has multiple effects on cancer therapy. It improves dendritic cell antigen presentation, which helps T lymphocytes recognize cancer cells. The capacity of NK cells and macrophages to destroy cancer cells is increased by IFN-α, which also increases their cytotoxic activity. Furthermore, IFN-α has direct antiproliferative effects on cancer cells, inhibiting their growth and replication [108].

#### Mechanism of action

IL-2 and IFN-α work in different ways, but they both improve therapy by increasing the ability of the immune system to detect and destroy cancer cells. IL-2 primarily acts by stimulating T-cell and NK cell proliferation and activation, whereas IFN-α enhances immune surveillance and antitumour immunity through multiple pathways, including increased cytotoxicity and inhibition of tumor proliferation. IL-2 has been utilized in the treatment of metastatic renal cell carcinoma and metastatic melanoma. In metastatic renal cell carcinoma, IL-2 therapy has demonstrated durable responses in a subset of patients, particularly those with good performance status and limited disease burden. Similarly, IL-2 has shown efficacy in the treatment of metastatic melanoma, where it enhances immune-mediated tumor regression. IFN-α is used to treat many types of malignancies, such as melanoma and certain leukemias. IFN-α therapy has been utilized as an adjuvant treatment for melanoma to decrease the likelihood of recurrence after surgical removal. The ability of IFN-α to modulate immune responses and inhibit tumor cell proliferation makes it a valuable component of treatment regimens for certain hematologic malignancies as well [109, 110].

### Oncolytic viruses

Oncolytic viruses are a promising frontier in cancer therapy, leveraging the natural ability of viruses to selectively infect and destroy cancer cells while leaving healthy cells intact. This innovative approach involves the use of genetically modified or naturally occurring viruses that have been engineered to specifically target and replicate within cancerous tissues [111]. The mechanism of action of oncolytic viruses begins with their selective entry into cancer cells, facilitated by specific receptors or vulnerabilities present on the surface of these cells. Once inside the cancer cell, the virus hijacks the cellular machinery to replicate, leading to the production of viral progeny. As infected cancer cells are filled with newly synthesized viruses, they eventually burst (lyse), releasing viral particles that can infect neighboring cancer cells. This process not only directly destroys cancer cells but also stimulates an immune response against the tumor. A viral infection within the microenvironment of a tumor results in the production of threat indicators and viral antigens, which in turn alerts the immune system to recognize and destroy cancer cells located throughout the body [112, 113].

Several types of viruses, including adenoviruses, herpes simplex viruses (HSVs), reoviruses, and vaccinia viruses, have been investigated for their oncolytic potential. Each virus type has unique characteristics that can be exploited for therapeutic purposes, such as its ability to infect specific types of cancer cells or its potential for genetic modification to increase tumor specificity and efficacy. Oncolytic viruses have demonstrated encouraging outcomes in clinical studies for a range of malignancies, including pancreatic cancer and glioblastoma, among others. These viruses are often administered directly into the tumor or intravenously, and they may be used alone or in combination with other cancer therapies, such as chemotherapy or immune checkpoint inhibitors, to maximize treatment efficacy [114, 115]. Challenges in the field include optimizing viral delivery, minimizing potential side effects and overcoming immune responses that can clear the virus before it reaches all tumor sites. Current research is continuously improving the utilization of oncolytic viruses, intending to broaden their usage in many types of cancer and enhancing patient outcomes by leveraging the potential of viruses to combat cancer in a precise and efficient way [116].

#### Mechanisms of action of oncolytic viruses

Oncolytic viruses exert their anticancer effects through several key mechanisms of action that selectively target and destroy tumor cells while sparing normal tissues. First and foremost, these viruses are engineered or naturally evolve to preferentially infect cancer cells, often due to specific vulnerabilities in the tumor's cellular machinery or signaling pathways. Upon entering a cancer cell, the virus begins to replicate, exploiting the host cell's resources. This replication process culminates in cell lysis, leading to the release of new viral particles and the subsequent spread of the virus to neighboring cancer cells. As infected cells die, they also release tumor-associated antigens and proinflammatory cytokines, which help stimulate an immune response against the tumor. This immune activation is a critical aspect of oncolytic virotherapy; the presence of viral antigens can increase the visibility of the tumor to the immune system, prompting an attack not only on virus-infected cells but also on surrounding cancer cells that may not have been directly infected by the virus. Additionally, oncolytic viruses can induce immunogenic cell death (ICD), which further amplifies the immune response by promoting the maturation of dendritic cells and the activation of T cells. Furthermore, oncolytic virotherapy can be effectively combined with other treatments, such as checkpoint inhibitors or other immunotherapies, to enhance the overall antitumor effect, thereby maximizing therapeutic outcomes. Overall, the multifaceted mechanisms of oncolytic viruses make them promising approaches in the landscape of cancer immunotherapy, leveraging both direct oncolysis and immune system activation to achieve a more comprehensive attack on tumors [117]. 

### Bispecific Antibodies and Their Mechanism of Action

Bispecific antibodies (BsAbs) are engineered immunotherapeutic agents that possess the unique ability to bind simultaneously to two different antigens or epitopes. This dual targeting ability allows them to bridge distinct cellular populations, increasing their therapeutic efficacy in the treatment of various diseases, particularly cancer [118]. BsAbs are designed to engage both immune effector cells, such as T cells or natural killer (NK) cells, and tumor cells, thereby promoting immune-mediated attack on tumors. The primary mechanism of action for bispecific antibodies involves the recruitment and activation of T cells. By binding to a specific tumor-associated antigen (TAA) on the surface of cancer cells and simultaneously engaging the CD3 receptor on T cells, BsAbs effectively redirect T cells to the tumor site. This interaction activates T cells, prompting them to proliferate and release cytotoxic molecules such as perforin and granzymes, leading to the destruction of targeted cancer cells [119, 120]. For example, bispecific T-cell engager (BiTE) antibodies, such as blinatumomab, target CD19 in B-cell malignancies and CD3 in T cells, demonstrating significant antitumor activity in diseases such as acute lymphoblastic leukemia (ALL) [121, 122]. In addition to T-cell engagement, some bispecific antibodies are designed to target both tumor antigens and immune checkpoint receptors, effectively combining direct tumor targeting with immune modulation. By inhibiting immune checkpoints, these BsAbs can further enhance T-cell activation and sustain antitumor responses.

Moreover, bispecific antibodies can be engineered to utilize different mechanisms of action depending on their structure and design. For example, some BsAbs can engage NK cells, macrophages, or other immune cells to exert their effects [123]. This versatility allows for tailored approaches in therapy, providing opportunities to enhance immune responses against various tumor types. Despite their potential, the clinical application of bispecific antibodies is not without challenges. Issues such as manufacturing complexity, the potential for immunogenicity, and the need to optimize dosing regimens require ongoing research and development [124, 125]. Nonetheless, bispecific antibodies represent a promising frontier in cancer immunotherapy, leveraging the power of the immune system to achieve targeted and effective antitumor responses.

## Recent advances in delivery modalities for cancer immunotheraphy

**Fig. 5:**
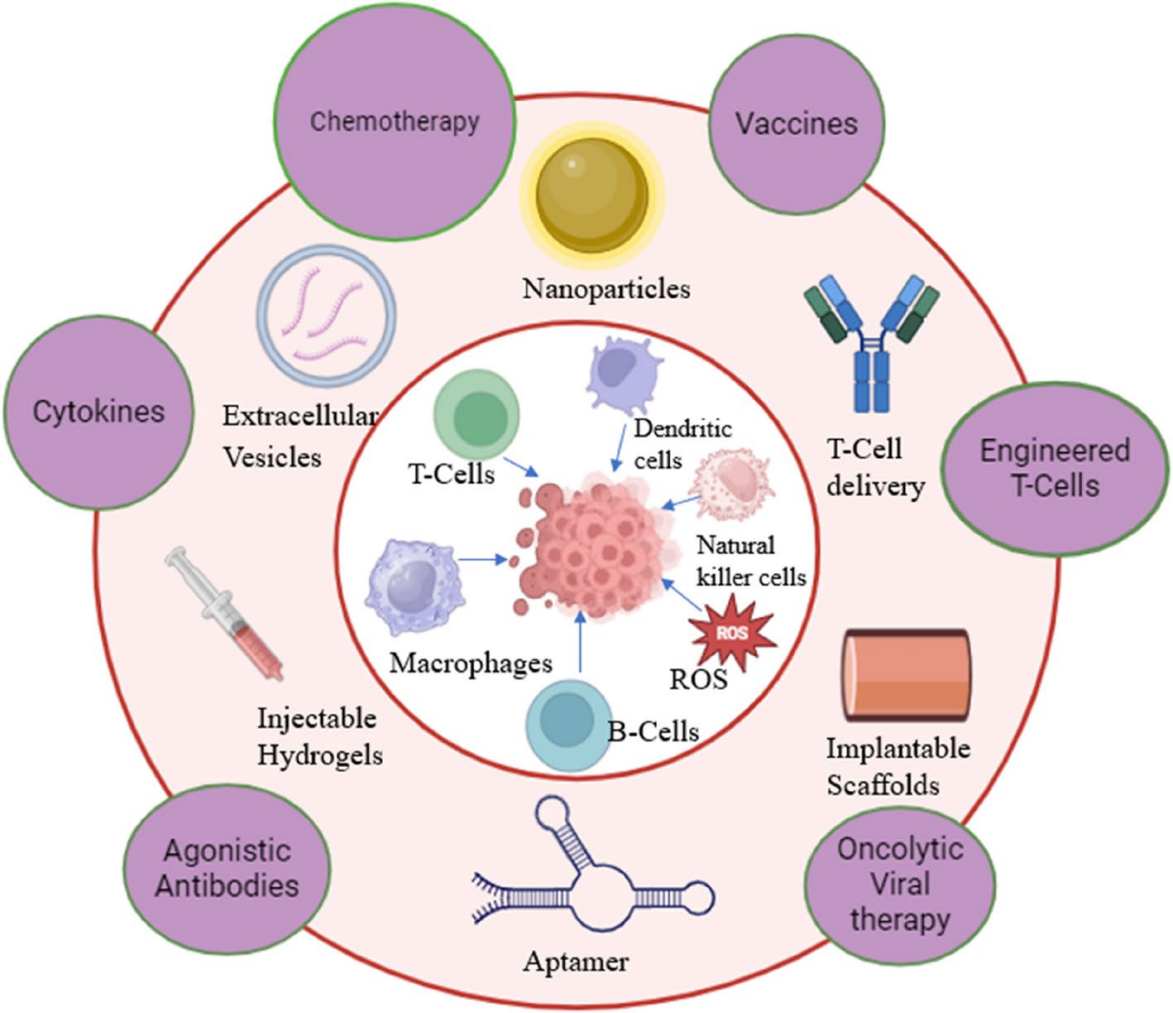
Multifaceted Approaches in Cancer Immunotherapy

The development of cancer immunotherapies that are both effective and safe for host cells depends on the development of delivery systems that are both efficient and effective (Figure 5). When developing a cancer treatment, it is necessary to consider a wide range of factors, including dosing, the manufacturing process, homing, degradation, and delivery. Physiochemical and biological factors should be considered throughout the drug development and delivery process. Table 1 shows the different ways in which cancer immunotherapy medicines can be delivered.

**Table 1:** Delivery techniques for cancer immunotherapy [126]

**Delivery Technology**	**Types/Source**	**Cargo**	**Cancer Type**	**Reference**
	**Liposomes**	ErbB2/HER2 peptide	Renal carcinoma	[127]
		OVA	Thymoma	[128]
		ACT-cell-specific antibodies and Interleukin-2 (IL-2)	Melanoma	[129]
		Plasmid encoding telomerase-specific oncolytic adenovirus	Colorectal cancer	[130]
	**Polymer**	OVA and Hydroxychloroquine	Thymoma	[131]
		PLK1 inhibitor and PD-L1 antibody	NSCLC	[132]
		IR780 and PD-L1 antagonist	Colorectal cancer	[133]
**Nanoparticles**	**Dendrimer**	PD-L1 siRNA and IL-2 encoding plasmid DNA	HCC	[134]
	**Inorganic nanocarriers**	Vesicular stomatitis virus	Colorectal cancer	[135]
		Adenovirus	Pancreatic cancer, Colorectal cancer	[136]
		mRNA-encoding OVA and R848	Melanoma	[137]
	**RNA/DNA Technology**	Anti-PD-1 antibody and CpG oligodeoxynucleotides	Melanoma	[138]
		OVA	Melanoma	[139]
	**Nanovaccine**	Peptide neoantigen (Adpgk) and R848 and CpG	Colorectal cancer	[140]
		cyclic dimeric guanosine monophosphate (CDG)	Melanoma	[141]
		Heterologous administration of HPV16 E7 epitope-loaded nanocomplexes inhibits tumor growth in mouse model	Melanoma	[142]
**Extracellular Vesicles**	**Dendritic cells**	VEGF siRNA	Breast cancer	[143]
	**Bone Marrow-Derived MSC**	TRAIL	Lung Cancer	[144]
	**A549 Lung Carcinoma cells (Human)**	Doxorubicin	Lung carcinoma	[145]
	**B16-F10 melanoma cells (Mouse)**	CpG DNA	Melanoma	[146]
	**H22 Hepatocarcinoma cells (Mouse)**	Doxorubicin, 5-FU	Hepatocarcinoma	[147]
	**Exosomes**	Let-7a miRNA	Breast cancer	[148]
		EGFR nanobodies	Epidermal	[149]
		Cisplatin	Ovarian cancer, Hepatocarcinoma	[150]
**Implantable Scaffolds**	**Collagen and HA cross-linking scaffold**	GEM, poly(I)	Breast cancer	[151]
	**PLG scaffold**	GM-CSF, CpG-ODNs	Melanoma	[152]
	**Hyaluronic acid scaffold**	CAR-NK cells	Breast cancer	[153]
**Injectable Scaffolds**	**Alginate Hydrogel**	Celecoxib, PD-1 antibody	Melanoma, Breast cancer	[154]
	**PEGylated poly(L-valine) hydrogel**	TCL, poly(I)	Melanoma	[155]
	**ROS-degradable hydrogel**	GEM, PD-L1 antibody	Melanoma, Breast cancer	[156]
**Cell-Based Delivery**	**Erythrocyte**	Curcumin	Liver cancer	[157]
		Glucose oxidase, Tirapazamine	Colon cancer	[158]
		DOX	Lymphoma	[159]
	**Cytotoxic T cells**	Taxol	Gastric cancer	[160]
	**NK cell**	TCPP	Breast cancer	[161]
	**Car-T Cells**	-	Glioblastoma, hepatic colorectal metastases, peritoneal carcinomatosis, pleural mesothelioma, mesothelioma	[162]

### Nanoparticle-based cancer immunotherapy

As useful additives in cancer immunotherapy, nanomaterials provide several advantages, such as a high surface-to-volume ratio, photodynamics, electrical and magnetic conductivity, optical absorption, and fluorescent behavior [163]. The application of nanomedicine-based drug delivery systems has attracted interest because of their biocompatibility, drug transport capabilities, ability to offer continuous drug release in cancer immunotherapy techniques, and current technological breakthroughs in nanoparticles [164]. A robust delivery platform that can traverse the intricate structure around the tumor is necessary to circumvent the obstacles to drug delivery to the tumor microenvironment [165]. Novel approaches to cancer immunotherapy, such as drug delivery via nanoparticles, hold great promise [166].

Researchers are exploring methods to provide therapies to tumors, as nanoparticles can selectively target cancer cells [167]. Enhancing the distribution and localization of medication within tumors can be accomplished by employing nanoparticle-based delivery systems that target tumors [168]. Exosomes, polymers, dendrimers, and inorganic nanocarriers are among the many platforms that nanoparticles use to transport proteins, inflammatory compounds, peptides, and small molecules [169].

The duration of drug accumulation in the tumor microenvironment is controlled by a crucial nanoparticle characteristic called enhanced permeability and retention (EPR) [170]. The ability of the immune system to target cancer cells is diminished when tumor-associated antigens elicit an immunological response with reduced anticancer effectiveness. When combined with a nanodelivery system, they are efficiently protected from degradation and can connect with antigen-presenting cells. This relationship stimulates cytotoxic T cells, which possess a potent anticancer function.

Copolymer micelles have demonstrated enhanced permeability and retention (EPR) effects when employed for tumor targeting. The copolymerization of polylactic acid and carboxymethyl cellulose, followed by modification with an anti-EpCAM antibody, can be utilized to selectively deliver the chemotherapeutic drug doxorubicin to hepatic cells (HepG2). The chemical doxorubicin was specifically released at the tumor location, and the modified drug-loaded micelles exhibited anticancer properties in both laboratory and live circumstances [171].

Nanofibers made of polylactic-co-lactic glycolic acid enhanced apoptosis and suppressed colon cancer, according to research by Badrinath et al. [172]. A study conducted by Chiang et al. demonstrated the successful eradication of tumors in a mouse model of 4T1 breast cancer via the use of a combination of anti-PDL1 checkpoint inhibitors and T-cell-activating drugs. This allows them to activate immune cells and eliminate tumors. IO@FuDex3 nanocomplexes were formed via conjugation with functionalized fucoidan-dextran and superparamagnetic iron oxide nanoparticles [173]. One further approach to target tumors involves the use of magnetic nanoparticles. This can be achieved in several ways, including changing the tumor environment, activating antigen-presenting cells (APCs), polarizing macrophages, stimulating T cells, and delivering natural killer (NK) cells [174].

A different study revealed that by combining a fluoropolymer with the antigen ovalbumin, dendritic cells can mature and present antigens more effectively, leading to tumor suppression. Cancer recurrence and metastasis are prevented by combining these fluoropolymers with antigens obtained from primary cancer cell membranes that have been surgically removed [175]. In their study on colon cancer and melanoma, Luo et al. demonstrated that neoantigen-based immunotherapy was effective and had visible effects. Researchers reported that nanovaccination effectively inhibited the growth of tumors and increased survival rates in a live animal model. In their study, Luo et al. reported that neoantigen-based immunotherapy effectively treated colon cancer and melanoma, and it also had observable effects. Researchers reported that nanovaccination effectively inhibited the growth of tumors and increased the rate of survival in a live animal model [176].

Various techniques have been employed to combine inorganic and organic nanoparticles to create highly efficient photothermal substances for the eradication of cancer cells [177, 178]. Gold nanoparticles were initially utilized for nanoparticle manufacturing because of their biocompatibility and optical characteristics. However, their limited effectiveness in photothermal therapy has led to the modification of their surface by coating them with silica. In laboratory studies, the resulting cluster of silica-coated gold nanoparticles effectively promoted photothermal transcription in prostate cancer cells. Within fifteen days, the malignancies had completely disappeared [179]. Alternatively, the immune system's response to cancer could be enhanced by the use of micrometal organic frameworks (MOFs) loaded with anti-DEC205 antibodies. In this investigation, sonodynamic immunotherapy was used to functionalize AMR-MOF@AuPt via ultrasonic-based deep-tissue-penetrating sonication. As a result, a substantial amount of reactive oxygen species are produced, leading to the successful elimination of cancerous cells and distant metastases [180].

### Biomaterial-based cancer immunotherapy

Biomaterial-based delivery systems are characterized by low invasiveness, precise targeting, regulated release, strong effectiveness, activation of immune cells, and low toxicity. These traits make them a promising strategy for cancer immunotherapy [172, 181]. To generate anticancer activity, implantable and injectable biomaterials based on scaffolds and nanomaterials are commonly used [182].

A cancer vaccine has been shown to increase the immune response against tumors by reducing immune suppression in the tumor microenvironment. This is achieved via the use of tumor lysate-based antigens, nano adjuvants that express Toll-like receptor (TLR 3 agonist), and gemcitabine, which eliminates myeloid-derived suppressor cells (MDSCs) [151]. Ren et al. employed a methacrylate hyaluronic acid implantable scaffold that is biodegradable and changeable in terms of its macroporous structure. Many chemicals—PTX, an antigen-presenting cell activator, R837, and compounds that inhibit immunological checkpoints—have been added to this scaffold. After that, a mouse model with 4T1 breast tumors was used to implant this scaffold. Increased numbers of antigen-presenting cells (APCs), decreased numbers of myeloid-derived suppressor cells (MDSCs), and increased numbers of M2 macrophages are among the outcomes of the immune response against cancer [183]. A three-dimensional synthetic hyaluronic acid scaffold was created by Ahn et al. This scaffold improved the efficacy against breast cancer in a surgically removed tumor model by increasing mRNA production, cytokine release, and tumor elimination [153].

Because different biomaterials have varied strengths in different contexts, the choice between injectable and implantable designs is often based on the specific requirements of the application. A less invasive alternative to surgical implantation is the direct injection of implantable materials into tissues or organs. This technique lessens the inflammatory response and tissue damage that are common in wounds [184]. Cryogels, hydrogels, and self-assembling systems are only a few examples of the natural and synthetic components that can be used to create injectable biomaterials [185]. A supramolecular hydrogel was created by Liu et al., which can be used as both an ICD and a therapeutic agent for immune checkpoint inhibitors. It is designed to be delivered locally and has dual activity [186].

### Extracellular vesicles

Extracellular vehicles (EVs) are small membranous structures that result from the fusion of the plasma membranes of cells and endosomes inside the cell. These structures are then released into the extracellular space. Every day, studies are uncovering the potential of EVs in delivering medications specifically to cancer cells, making them a promising drug delivery technique [187, 188]. Complex membranous EVs preferentially enter cells by passing via tight junctions [189]. To target a liver malignancy, Wang et al. used exosomes as a delivery mechanism. Because of its poor solubility, PTX has limited therapeutic efficacy; nonetheless, it has been incorporated into exosomes to increase its potential. With this method, tumor preservation and inhibition were both shown to be more effective [190]. The production of exosomes by dendritic cells with cancer antigens and functional major histocompatibility complexes (MHCs) was reported by Zitvogel et al. These findings revealed that these exosomes could initiate the neutralization of tumors by cytotoxic T lymphocytes (CTLs) [191]. Researchers have used curcumin-loaded exosomes to treat malignant glioma in brain tissue in a live mouse model by penetrating the blood‒brain barrier [192].

#### T-cell Therapy Delivery Methods

Recent developments in technology have made it possible to isolate, genetically modify, and cultivate T cells externally from a patient's blood via T-cell-based treatments at the top of the field of cancer immunotherapy [193]. By modifying T-cell receptors and introducing cancer-infiltrating lymphocytes (TILs), the body can produce particular antigens and HLAs, which destroy cancer cells [194].

With respect to blood cancers, adoptive cell therapy—specifically T-cell therapy—is second to none. Patients in the US can now receive therapy with CAR-T cells, a therapy that has recently received multiple FDA approvals, for a range of B-cell malignancies. This therapy involves collecting patient blood and modifying T cells to specifically target and treat these types of cancers. While this technique has proven to be effective against blood cancers, its efficacy against solid tumors is limited owing to inadequate penetration into the intricate tumor microenvironment. As a result, researchers are always looking for a better way to deliver CAR-T-cell therapies to cancer cells [195]. Local injection or implanted bioscaffold administration has been successful, and long-term distribution inside the cancer microenvironment has improved with parallel administration of CAR-T cells with immunostimulatory molecules. To demonstrate this point, a mouse model was used by Grosskopf AK et al. to administer IL-15-loaded CAR-T cells via a polymer‒nanoparticle (PNP) hydrogel. This method allows for the delivery of cells both near and far from tumors, potentially enabling the treatment of solid tumors and achieving a cure [196].

Moreover, employing multiple treatment techniques may improve the efficacy of solid tumor treatment by leveraging a synergistic effect. Hu et al. investigated the utilization of CAR-T cells in combination with immune checkpoint inhibitors in a murine model of melanoma. The biodegradable hydrogel encapsulates CAR-T cells and specifically targets human chondroitin sulfate proteoglycan 4 (CSPG4.CAR). The hydrogel is covered with nanoparticles that contain IL-15 and anti-PDL1. These nanoparticles are linked to human platelets. When anti-PDL1 and anti-IL-15 agents are administered together, the former can stimulate the development of CAR-T cells, and the latter can block the PD1/PDL1 pathway, allowing for the effective elimination of cancer cells [197]. Several efficient delivery methods have been developed for the administration and enhancement of CAR-T-cell accessibility to solid tumors. Surgically inaccessible or incompletely removed tumors can be precisely and efficiently targeted with biomedical polymeric devices developed through engineering. A complicated technique including the combination of soluble biomolecules with ligands for T-cell activation antibodies can be employed to achieve antitumor action against cancer cells. T lymphocyte migration to tumor sites was enhanced by three-dimensional bioscaffolds, such as polymerized alginate-collagen mimetic peptide matrices. This effect was also observed when porous silica microparticles were combined with the matrices, allowing for the encapsulation and controlled release of biomolecules over a prolonged period of time [198].

## Personalized Immunotherapeutic Strategies Based on Unique Genetic and Molecular Tumor Profiles

Personalized cancer immunotherapy is an evolving frontier in oncology, enabling more effective, targeted, and safer treatment options on the basis of the molecular fingerprints of individual tumors. The following sections describe how such personalized strategies are tailored across major cancer types.

### Melanoma: Neoantigen burden and TIL profiling

Melanoma is one of the most immunogenic cancers due to its high tumor mutational burden (TMB), which often results from ultraviolet radiation-induced DNA damage. This high mutational load creates numerous tumor-specific neoantigens that are easily recognized by cytotoxic T lymphocytes. Immune checkpoint inhibitors, such as nivolumab and pembrolizumab, are particularly effective in patients with elevated TMB and high PD-L1 expression. Additionally, the presence of TILs, especially CD8+ T cells, is a strong predictor of the immunotherapy response. Personalized vaccines based on tumor neoantigens and adoptive TIL therapies are also being developed. These therapies rely on sequencing the patient’s tumor and identifying dominant T-cell clones for ex vivo expansion and reinfusion, enabling a customized attack on tumor-specific targets [199, 200].

### Non-small cell lung cancer (NSCLC): TMB and STK11/KEAP1 mutation profiling

In NSCLC, personalized immunotherapy is guided by both mutational load and actionable genetic alterations. High TMB and MSI-H status are linked with better responses to PD-1/PD-L1 blockade. However, the presence of STK11 and KEAP1 mutations, which commonly cooccur with KRAS mutations, can negatively impact immune cell infiltration and responsiveness to immunotherapy. On the other hand, EGFR and ALK mutations often predict poor outcomes with ICIs, prompting the use of targeted therapies first. Molecular testing via next-generation sequencing (NGS) has become standard practice, helping clinicians decide whether to administer ICIs alone or in combination with chemotherapy or delay their use. Ongoing studies are also exploring gene expression-based immune signatures and the role of epigenetic modifiers in sensitizing NSCLC tumors to immunotherapy [201, 202].

### Colorectal Cancer (CRC): MSI-H/dMMR as a biomarker for PD-1 inhibitors

Colorectal cancer is one of the most well-established models of molecularly guided immunotherapy. Tumors that are microsatellite instability-high (MSI-H) or mismatch repair-deficiency (dMMR) exhibit high neoantigen loads and robust immune cell infiltration, making them ideal candidates for PD-1 inhibitors such as pembrolizumab. However, the majority of colorectal cancers are microsatellite stable (MSS) and immunologically "cold." Personalized strategies for these patients include combining ICIs with chemotherapy, anti-VEGF agents, or even gut microbiota modulation to improve immune responsiveness. Additionally, emerging evidence suggests that immune checkpoint efficacy can be enhanced through fecal microbiota transplantation or probiotic supplementation to restore microbial balance and immune activation in the tumor microenvironment [203, 204].

### Breast cancer: Subtype-specific immune strategies

Breast cancer is a heterogeneous disease, and the role of immunotherapy is most prominent in triple-negative breast cancer (TNBC). TNBC is often associated with increased mutational burden, increased PD-L1 expression, and increased presence of TILs, all of which are predictive markers for immunotherapy response. PD-L1 testing is routinely used to determine eligibility for therapies such as atezolizumab or pembrolizumab in TNBC patients. In contrast, hormone receptor-positive and HER2-positive subtypes are typically less immunogenic. However, molecular profiling of HER2+ cancers may reveal the eligibility of HER2-targeted CAR-T-cell therapies or bispecific antibodies. Additionally, BRCA1/2-mutated tumors may benefit from combined PARP inhibitors and immune checkpoint blockade, offering a personalized approach based on DNA repair deficiency [205, 206].

### Prostate Cancer: Low Immunogenicity and DNA Repair Defects

Prostate cancer has traditionally been considered immunologically “cold,” exhibiting a low TMB and minimal T-cell infiltration. However, a subset of patients with DNA damage repair (DDR) gene mutations—particularly mutations in BRCA1, BRCA2, and ATM—can benefit from PARP inhibitors and potentially from combination therapy with ICIs. Furthermore, the presence of AR-V7 splice variants in circulating tumor cells can influence the responsiveness to hormonal therapy versus immunotherapy. Sipuleucel-T, an autologous dendritic cell vaccine targeting prostatic acid phosphatase (PAP), exemplifies the personalization of immunotherapy in prostate cancer. Liquid biopsies are now being used to track androgen receptor mutations and other genomic changes, guiding personalized treatment plans in patients with metastatic castration-resistant prostate cancer (mCRPC) [207].

### Glioblastoma: Personalized Neoantigen Vaccines and IDH Mutations

Glioblastoma (GBM) remains one of the most challenging cancers to treat with immunotherapy because of its immunosuppressive microenvironment and low TMB. However, molecular subtyping and mutation profiling offer opportunities for personalization. For example, tumors with IDH1 mutations may respond differently to immunotherapy than IDH1 wild-type tumors. Neoantigen-based peptide vaccines tailored to mutations such as EGFRvIII have shown early promise. Additionally, CAR-T cells targeting IL13Rα2 and HER2 are being explored in clinical trials, particularly when supported by tumor HLA typing and antigen expression profiles. Despite the ability of the blood‒brain barrier to limit immune cell access, novel delivery platforms and localized immunotherapy (e.g., intratumoral injections) are being developed to improve effectiveness in GBM patients [208].

### Ovarian cancer: Immune subtypes and BRCA mutations

Ovarian cancer presents a moderate mutational burden but has subgroups that can benefit from personalized immunotherapy. BRCA1/2-mutated tumors show increased DNA damage signaling and may respond to PARP inhibitors in combination with ICIs. Tumors can also be categorized by immune gene signatures into immune-infiltrated or immune-desert subtypes, influencing the therapeutic response. HLA typing and neoantigen prediction have been used to design personalized dendritic cell vaccines and TCR-engineered T cells that target tumor antigens such as NY-ESO-1 or WT1. Additionally, the levels of cytokines, such as CXCL9 and CXCL10, and PD-L1 expression are being studied as biomarkers to stratify patients for immune-based therapies in advanced or platinum-resistant ovarian cancer [209].

## Predictive Biomarkers for Stratifying Immunotherapy Responders

The identification of reliable predictive biomarkers is critical for optimizing patient selection, improving treatment efficacy, and minimizing unnecessary toxicity in cancer immunotherapy. Since immune checkpoint inhibitors and adoptive cell therapies do not benefit all patients equally, biomarkers help stratify those most likely to respond and guide personalized treatment strategies across various cancer types. One of the most widely studied biomarkers is PD-L1 expression on tumor and immune cells. High PD-L1 expression has been associated with improved response rates to anti-PD-1/PD-L1 agents in NSCLC, urothelial carcinoma, and triple-negative breast cancer. However, PD-L1 expression is heterogeneous, dynamic, and influenced by prior treatments, limiting its utility as a sole predictor [210, 211]. TMB is another important biomarker reflecting the number of somatic mutations in a tumor. High TMB often correlates with increased neoantigen production, making tumors more recognizable to T cells [212, 213]. Clinical trials, such as CheckMate 227, have shown that high TMB is associated with superior responses to nivolumab + ipilimumab in patients with NSCLC. TMB is also used as a tumor-agnostic biomarker for pembrolizumab in the FDA-approved indication for TMB ≥10 mutations/Mb [214]. The microsatellite instability-high (MSI-H) or deficient mismatch repair (dMMR) status serves as a robust predictive biomarker, especially in colorectal, endometrial, and gastric cancers. MSI-H tumors are immunologically "hot," often presenting with increased T-cell infiltration and neoantigen load. Pembrolizumab is FDA-approved for MSI-H/dMMR solid tumors, regardless of their tissue origin [215].

In addition to genomic markers, immune gene expression profiles, such as interferon-gamma (IFN-γ)-related signatures, have shown predictive value in patients with melanoma and renal cell carcinoma. Similarly, the density of TILs, especially CD8⁺ T cells at the invasive margin, is correlated with favorable immunotherapy responses in patients with melanoma and ovarian cancer [216]. Circulating biomarkers such as ctDNA dynamics, soluble PD-L1, and exosomal RNA offer minimally invasive methods to monitor therapy response and resistance in real time. For example, early decreases in ctDNA levels after immunotherapy initiation are associated with longer progression-free survival. HLA typing and T-cell receptor (TCR) repertoire diversity are also emerging tools for assessing the likelihood of successful antigen presentation and immune activation. Patients with diverse TCR clonotypes tend to mount stronger responses to checkpoint blockade [217]. Table 2 shows the predictive biomarkers for immunotherapy response for all types of cancer.

Finally, microbiome composition is being explored as a novel predictive biomarker, with studies showing that the presence of specific gut bacteria enhances the efficacy of PD-1 blockade, particularly in melanoma and colorectal cancer. Together, these biomarkers form the foundation for precision immuno-oncology, allowing clinicians to tailor treatment decisions, identify nonresponders early, and improve overall patient outcomes.

**Table 2:** Predictive biomarkers for immunotherapy response across cancer types

**Biomarker**	**Cancer Type(s)**	**Predictive Role/Significance**	**Associated Drugs/Immunotherapies**
PD-L1 expression	NSCLC, TNBC, melanoma, bladder, gastric	High PD-L1 correlates with improved response to PD-1/PD-L1 inhibitors	Pembrolizumab, Nivolumab, Atezolizumab, Durvalumab
Tumor Mutational Burden (TMB)	NSCLC, melanoma, bladder, cervical	High TMB predicts better neoantigen load and ICI response	Pembrolizumab (TMB ≥10 mutations/Mb), Nivolumab + Ipilimumab
Microsatellite Instability-High (MSI-H)/dMMR	Colorectal, endometrial, gastric, prostate	Strong predictor of response due to high immunogenicity	Pembrolizumab, Nivolumab
TIL density (CD8+ T cells)	Melanoma, ovarian, breast, cervical	High TIL infiltration correlates with better ICI response	General ICI predictor (not drug-specific)
IFN-γ–related gene signature	Melanoma, RCC	Indicates a preinflamed TME conducive to ICI response	Predictive of PD-1 blockade response
Circulating tumor DNA (ctDNA)	Lung, colorectal, breast, melanoma	Early decline during treatment correlates with clinical response	Dynamic response monitoring (nonspecific to drug)
HLA typing & TCR clonality	Melanoma, NSCLC	Predicts antigen presentation and T-cell recognition	TCR-T therapy, personalized vaccine selection
Gut microbiota composition	Melanoma, CRC, NSCLC	Specific bacteria (e.g., *Akkermansia*, *Bifidobacterium*) enhance ICI efficacy	Modulates PD-1/PD-L1 responses; studied with FMT
Beta-2 microglobulin (B2M) mutations	Melanoma, prostate	Associated with resistance to ICIs due to MHC-I loss	Used in resistance prediction, not drug-targeted
LAG-3 and TIM-3 expression	Melanoma, HNSCC, gastric	Emerging markers of immune exhaustion and ICI resistance	Under investigation with dual checkpoint inhibitors

## Combination Immunotherapy Strategies to Enhance Efficacy and Overcome Resistance

While immune checkpoint inhibitors and adoptive cell therapies have transformed cancer treatment, their effectiveness is limited in a significant proportion of patients due to primary or acquired resistance. Rational combination therapies have emerged as a potent strategy to overcome these barriers. By integrating immunotherapy with conventional and novel modalities, including chemotherapy, targeted therapy, radiotherapy, and microbiota modulation, researchers aim to increase tumor immunogenicity, improve immune cell infiltration, and sustain durable antitumor responses.

### Immunotherapy Plus Chemotherapy

Chemotherapy can modulate the immune response by inducing ICD, increasing tumor antigen release, and reducing the number of immunosuppressive cells, such as regulatory T cells (Tregs) and MDSCs. Combining chemotherapy with immune checkpoint inhibitors has shown significant clinical benefit in several cancers. In NSCLC, the KEYNOTE-189 trial demonstrated that, compared with chemotherapy alone, combining pembrolizumab with platinum-based chemotherapy significantly improved overall survival [218]. Similarly, in TNBC, the IMpassion130 trial revealed that atezolizumab plus nab-paclitaxel improved progression-free survival (PFS) in PD-L1-positive patients [219]. In gastric cancer, the CheckMate 649 study evaluated nivolumab combined with oxaliplatin-based chemotherapy, which resulted in improved overall survival and a higher response rate in patients with a PD-L1 combined positive score (CPS ≥5). These results support the synergy between chemotherapy and immune checkpoint blockade across multiple solid tumors [220].

### Immunotherapy Plus-Targeted Therapy

Targeted therapies against tumor-specific oncogenes (e.g., EGFR, BRAF, and VEGF) can alter the tumor microenvironment, enhancing immunogenicity and responsiveness to immunotherapy. One mechanism involves reducing VEGF-mediated immunosuppression and enhancing dendritic cell maturation. In renal cell carcinoma (RCC), the combination of avelumab (PD-L1 inhibitor) and axitinib (VEGFR inhibitor) led to superior PFS and OS compared with sunitinib monotherapy in the JAVELIN Renal 101 trial [221]. Another example is the CLEAR study, which reported that lenvatinib plus pembrolizumab extended PFS to 23.9 months compared with 9.2 months with sunitinib [222]. In melanoma, the COMBI-i trial investigated spartalizumab (PD-1) with dabrafenib and trametinib (BRAF/MEK inhibitors). Although not statistically significant overall, subgroup analysis suggested a benefit in certain molecular phenotypes with higher T-cell infiltration. Preclinical models also show that BRAF inhibitors increase antigen expression and immune infiltration, which can be exploited in personalized treatment plans [223]. In hepatocellular carcinoma, combining atezolizumab with bevacizumab (an anti-VEGF monoclonal antibody) was shown in the IMbrave150 trial to significantly improve OS and PFS over sorafenib monotherapy, reinforcing the concept that VEGF blockade can synergize with immune activation [224].

### Immunotherapy plus radiotherapy

Radiation therapy not only causes tumor cell death but also enhances antigen presentation, increases MHC class I expression, and promotes the infiltration of immune cells into the tumor. These effects contribute to the abscopal effect, in which radiation at one site triggers immune-mediated regression at distant, unirradiated sites. A pivotal example is a phase II trial in metastatic NSCLC (the PEMBRO-RT study), where patients received pembrolizumab alone or after stereotactic body radiotherapy (SBRT) to a single lesion. The response rate nearly doubled in the radiotherapy plus pembrolizumab arm [225]. In melanoma, case reports and small trials have documented synergistic responses when combining anti-CTLA-4 (ipilimumab) with localized radiotherapy. Radiation acts as an in situ vaccine by releasing tumor antigens, enhancing systemic antitumor immunity. Ongoing trials are evaluating the optimal dosing, fractionation, and sequencing of radiotherapy with ICIs to optimize outcomes in patients with solid tumors such as head and neck cancer, prostate cancer, and glioblastoma [226].

### Immunotherapy and Microbiota Modulation

The gut microbiome profoundly influences systemic immune responses and has emerged as a critical determinant of immunotherapy efficacy. Specific commensal bacteria have been linked to enhanced responses to checkpoint inhibitors, whereas dysbiosis often caused by antibiotic use is correlated with poor outcomes.

A landmark study by Routy et al. revealed that patients with NSCLC or RCC who had taken antibiotics had significantly reduced responses to PD-1 inhibitors. The presence of Akkermansia muciniphila and Faecalibacterium prausnitzii was associated with a restored ICI response in preclinical models [227]. In a phase I clinical trial, fecal microbiota transplantation (FMT) from ICI responders to ICI-resistant melanoma patients led to restored clinical responses in 30% of participants. Changes in microbial composition after FMT are correlated with increased T-cell activation and decreased MDSC populations [228]. Prebiotic and probiotic strategies are also being evaluated. In murine models, the administration of Bifidobacterium breve improved anti-PD-L1 efficacy through increased dendritic cell activation and CD8⁺ T-cell infiltration. Clinical trials are ongoing to assess the effects of dietary fiber, polyphenols, and live biotherapeutics in combination with immunotherapy [229].

#### Role of the Gut Microbiota in Enhancing Colorectal Cancer Immunotherapy

CRC, especially its MSS form, presents a major therapeutic challenge because of its poor response to immune checkpoint inhibitors (ICIs). Recent studies have revealed that the gut microbiota, which comprises trillions of commensal microorganisms, plays a pivotal role in regulating systemic immune responses and modulating tumor behavior. Modifying the microbiota has emerged as a promising strategy to increase the effectiveness of immunotherapy, especially when it is used in combination with other modalities, such as chemotherapy or immune checkpoint blockade [230].

Certain bacterial taxa, such as Akkermansia muciniphila, Faecalibacterium prausnitzii, and Bifidobacterium longum, have been linked to improved antitumor immunity and better responses to ICIs in patients with CRC. These bacteria promote antigen presentation, enhance T-cell infiltration, and modulate cytokine profiles to favor an immunostimulatory TME [231]. For example, A. muciniphila enhances dendritic cell maturation and increases IL-12 production, thereby priming CD8+ cytotoxic T cells to recognize tumor antigens more effectively [232].

In a pivotal preclinical study, fecal microbiota transplantation (FMT) from ICI-responsive individuals into germ-free or antibiotic-treated mice with CRC significantly restored the response to anti-PD-1 therapy. These findings were translated into early-phase clinical trials. For example, a phase I trial demonstrated that combining FMT with nivolumab led to partial responses in immune-refractory metastatic melanoma patients, and similar trials are underway in MSS-CRC patients [233].

In addition to checkpoint blockade, chemotherapy and microbiota-targeted approaches show promise. Oxaliplatin and 5-FU can disrupt the intestinal barrier and microbiota composition, but when coadministered with microbiota-supportive agents (e.g., dietary fiber, prebiotics, or probiotic supplements), this negative impact may be reversed. These combinations enhance T-cell infiltration and reduce regulatory T cells in the tumor microenvironment [234]. Furthermore, novel synbiotics (prebiotic + probiotic combinations) and postbiotics (microbial metabolites such as butyrate or inosine) are under development to increase gut-immune signaling. Some of these agents can increase tumor MHC-I expression and T-cell priming, potentiating the efficacy of ICIs [235]. Overall, the integration of microbiota modulation into CRC treatment particularly for MSS tumors has immense potential to convert immunologically “cold” tumors into “hot,” therapy-responsive phenotypes. Future precision medicine approaches may involve personalized microbiome profiling, enabling the use of tailored microbial interventions alongside conventional or immunotherapy.

### Emerging Combinations: Epigenetic and Metabolic Modulators

Novel agents targeting epigenetic regulators (e.g., HDAC inhibitors and DNA methyltransferase inhibitors) are being studied to reverse immune evasion. For example, entinostat, an HDAC inhibitor, increases MHC expression and the antigen processing machinery, sensitizing tumors to ICIs [236]. In ER-positive breast cancer, a phase II study of entinostat and atezolizumab demonstrated enhanced immune activation and preliminary clinical benefit. Similarly, metformin and IDO inhibitors are under investigation for their ability to reprogram tumor metabolism and overcome T-cell exhaustion [237]. Furthermore, nanoparticle-based platforms enable the codelivery of immunomodulatory drugs and ICIs for improved pharmacokinetics and safety profiles. A preclinical study using PLGA nanoparticles loaded with PD-L1 siRNA and an IL-2 plasmid revealed complete tumor regression in hepatocellular carcinoma models [238]. Table 3 shows a summary of combination immunotherapy strategies with clinical and preclinical evidence.

**Table 3:** Summary of combination immunotherapy strategies with clinical and preclinical evidence

**Combination Strategy**	**Cancer Type(s)**	**Key Agents**	**Study Example**	**Outcome/Impact**
**Immunotherapy + Chemotherapy**	NSCLC	Pembrolizumab + platinum-based chemo	KEYNOTE-189 (NEJM, 2018)	Improved OS (22.0 vs. 10.7 months), higher response rates
	TNBC	Atezolizumab + nab-paclitaxel	IMpassion130 (NEJM, 2019)	Longer PFS in PD-L1+ TNBC patients (7.5 vs. 5.0 months)
	Gastric cancer	Nivolumab + oxaliplatin	CheckMate 649	Improved OS in CPS ≥5 population
**Immunotherapy + Targeted Therapy**	RCC	Avelumab + Axitinib	JAVELIN Renal 101	Increased PFS (13.8 vs. 8.4 months) and OS
	RCC	Lenvatinib + Pembrolizumab	CLEAR Study	PFS extended to 23.9 months
	Melanoma	Spartalizumab + BRAF/MEK inhibitors	COMBI-i Trial	Improved T-cell infiltration (modest clinical benefit)
	HCC	Atezolizumab + Bevacizumab	IMbrave150	OS: 19.2 vs. 13.4 months compared to sorafenib
**Immunotherapy + Radiotherapy**	NSCLC	Pembrolizumab + SBRT	PEMBRO-RT Study	Response rate doubled (41.7% vs. 19.2%)
	Melanoma	Ipilimumab + Radiotherapy	Case reports, early trials	Observed abscopal effect in metastatic lesions
**Immunotherapy + Microbiota Modulation**	Melanoma, NSCLC	PD-1 inhibitors + FMT	Baruch et al.,	Restored ICI responsiveness in 30% of ICI-resistant patients
	NSCLC, RCC	PD-1 inhibitors + commensal bacteria	Routy et al.,	*A. muciniphila* improved ICI outcomes; antibiotics impaired response
**Immunotherapy + Epigenetic/Metabolic Modifiers**	Breast cancer	Atezolizumab + Entinostat	Phase II trial	Increased MHC expression and TILs; early clinical benefit
	HCC (preclinical)	PD-L1 siRNA + IL-2 plasmid (NP delivery)	Liu et al.,	Complete tumor regression in mouse models

## Strategies to Modify the Tumor Microenvironment to Overcome Immunosuppression

The tumor microenvironment (TME) plays a pivotal role in dictating the success or failure of immunotherapeutic interventions. In many solid tumors, the TME is highly immunosuppressive and characterized by regulatory T cells (Tregs), myeloid-derived suppressor cells (MDSCs), tumor-associated macrophages (TAMs, often M2-polarized), hypoxia, and inhibitory cytokines such as TGF-β and IL-10. These components collectively inhibit cytotoxic T-cell activity, impair antigen presentation, and facilitate immune escape. Thus, reprogramming the TME to a more immunostimulatory state is a critical goal in improving immunotherapy outcomes. One strategy involves targeting immunosuppressive cellular populations. Inhibitors of CSF1R have been used to deplete or repolarize M2 macrophages into proinflammatory M1 macrophages, enhancing antigen presentation and T-cell activation. Clinical trials using cabiralizumab (anti-CSF1R) in combination with PD-1 inhibitors have shown early signs of efficacy in treating pancreatic and lung cancers. Similarly, blockade of CCR4 (using mogamulizumab) has been used to deplete Tregs and improve immune responsiveness [239, 240]. Similarly, blockade of CCR4 (using mogamulizumab) has been used to deplete Tregs and improve immune responsiveness [239, 240].

Another approach is the use of IDO1 inhibitors, which block tryptophan metabolism, which contributes to immune suppression and T-cell anergy. Although early trials (e.g., epacadostat + pembrolizumab) failed to meet endpoints, next-generation indoleamine 2,3-dioxygenase (IDO) inhibitors and dual pathway inhibitors (e.g., IDO1/TDO2) are under investigation. Modulation of the tumor vasculature is another critical avenue. Anti-angiogenic agents such as bevacizumab normalize abnormal blood vessels, increase T-cell infiltration, and reduce hypoxia. When combined with immune checkpoint inhibitors—as demonstrated in hepatocellular carcinoma (IMbrave150 trial)—this approach improves TME accessibility and reduces immune evasion [241]. Targeting metabolic pathways is also gaining attention. Tumor cells often outcompete immune cells for glucose and amino acids. Agents such as arginase inhibitors, glutaminase inhibitors, and lactate dehydrogenase (LDH) blockers aim to restore the metabolic fitness of effector T cells within the TME [242]. Nanotechnology is increasingly being leveraged to deliver TME-modulating agents directly to the tumor site. For example, nanoparticles carrying TGF-β inhibitors, siRNAs, or IL-12 plasmids have shown success in reducing immune suppression and enhancing cytotoxic T-cell infiltration in preclinical models of breast and pancreatic cancer [243]. Finally, radiation and oncolytic viruses are used to transform immune "cold" tumors into "hot" tumors by promoting local inflammation and antigen release. These therapies also upregulate danger signals and chemokines (e.g., CXCL9/10), facilitating T-cell recruitment and activation [244]. Together, these strategies aim to shift the TME from an immunosuppressive milieu to one that favors immune recognition and tumor elimination, ultimately improving the efficacy and durability of immunotherapeutic responses.

## Clinical Trials and Patents in Immunotherapy for Cancer Treatment

Immunotherapy, which uses the immune system of the body to fight tumors, is one of the most exciting new ways to treat cancer. Clinical trials form the backbone of evaluating the safety and efficacy of these innovative therapies across various cancer types, aiming to translate scientific discoveries into viable treatments. Concurrently, patents play a crucial role in protecting intellectual property related to novel immunotherapeutic agents and delivery technologies, fostering innovation and investment in this rapidly evolving field. Tables 4, 5 outline current clinical trials exploring diverse immunotherapeutic approaches and highlight key patents that underscore ongoing advancements in cancer immunotherapy.

**Table 4:** Clinical Trials and Patients in Immunotherapy for Cancer Treatment

**Delivery Technology**	**Therapy**	**Cancer Type**	**Clinical Trial Identifier**	**Reference**
Intravenous Infusion	Nivolumab (Opdivo) + Ipilimumab (Yervoy)	Non-Small Cell Lung Cancer (NSCLC)	NCT02477826	[245]
Intratumoral Injection	Talimogene laherparepvec (T-VEC)	Melanoma	NCT02263508	[246]
Oral Administration	Axalimogene filolisbac (ADXS-HPV)	Cervical Cancer	NCT01266460	[247]
CAR T-Cell Therapy	Axicabtagene ciloleucel (Yescarta)	Diffuse Large B-Cell Lymphoma	NCT02348216	[248]
mRNA Vaccine	mRNA-4157	Solid Tumors	NCT03313778	[249]
Oncolytic Virus	CG0070	Bladder Cancer	NCT02365818	[250]
Bite Antibody	Blinatumomab (Blincyto)	Acute Lymphoblastic Leukemia	NCT02013167	[251]
Peptide Vaccine	PVX-410	Multiple Myeloma	NCT01718899	[252]
Nanoparticle Delivery	nanocamptothecin CRLX101 with capecitabine	Various Solid Tumors	NCT02010567	[253]
Radiolabeled Antibody	Ibritumomab tiuxetan (Zevalin)	Non-Hodgkin's Lymphoma	NCT00514908	[254]

**Table 5:** Patents Related to Cancer Immunotherapy

**Patent Number**	**Title**	**Assignee**	**Summary**
US10072132B2	Anti-PD-1 Antibody	Merck Sharp & Dohme Corp.	This patent describes to pembrolizumab (Keytruda), which is an anti-PD-1 monoclonal antibody employed in the treatment of several types of cancer.
US9821057B2	Anti-CTLA-4 Antibodies and Their Uses	Bristol-Myers Squibb Company	This patent involves ipilimumab (Yervoy), an anti-CTLA-4 antibody utilized in melanoma treatment.
US10300138B2	Compositions and Methods for Enhancing Anti-Tumor Immunity	Genentech, Inc.	Focuses on combination therapies involving checkpoint inhibitors and costimulatory molecules.
RU2017102769A	CAR T Cells Specific for EGFRvIII and Uses Thereof	Juno Therapeutics, Inc.	For glioblastoma, this patent talks about CAR-T cells that target EGFRvIII.
US7235641B2	Bispecific Antibodies for T-Cell Activation	Amgen Inc.	Covers bispecific T-cell engager (BiTE) antibodies that simultaneously target tumor cells and T cells.
AU2017210031B2	NK Cell Therapies	Fate Therapeutics, Inc.	Involves methods for producing and using natural killer (NK) cells for immunotherapeutic applications.
US20090010948A1	Anti-tumor vaccines delivered by dendritic cells devoid of interleukin-10	Fang Ping Huang, Yu Xiao Chen, Kwan Man	This patent describes anti-tumor vaccines delivered by dendritic cells that do not produce interleukin-10, which is an immunosuppressive cytokine.
US20040156846A1	Therapy via targeted delivery of nanoscale particles using L6 antibodies	Wolfgang Daum, Gerald DeNardo, Diane Ellis-Busby, Alan Foreman, Douglas Gwost, Erik Handy, Robert Ivkov	Covers targeted delivery of nanoscale particles to cancer cells using L6 antibodies.
WO2017151727A1	Enhanced cancer immunotherapy by microneedle patch-assisted delivery	Zhen GU, Chao Wang, Yanqi YE	Describes the use of microneedle patches to enhance the delivery of cancer immunotherapy agents.
US20160361268A1	Intralymphatic delivery of hyaluronan nanoparticle for cancer metastasis	Chih-Peng Liu, Ya-Chin Lo, Ming-Cheng Wei, Maggie LU, Shuen-Hsiang CHOU, Shih-Ta Chen, Hsiang-Wen TSENG	Discusses the use of hyaluronan nanoparticles for intralymphatic delivery to prevent cancer metastasis.
WO2011097384A2	Tumor targeted delivery of immunomodulators by nanoplymers	Dapeng Zhou, Li Chun, Patrick Hwu	Focuses on tumor-targeted delivery of immunomodulatory agents using nanopolymers.
US8785371B2	Drug delivery of temozolomide for systemic based treatment of cancer	Rameshwar Patil, Eggehard Holler, Keith L. Black, Julia Y. Ljubimova	This patent describes a method for the systemic delivery of temozolomide for treating cancer.
US20160346204A1	Nanoscale carriers for the delivery or codelivery of chemotherapeutics, nucleic acids, and photosensitizers	Wenbin Lin, Chunbai He, Demin Liu	Covers the use of nanoscale carriers for delivering chemotherapeutics, nucleic acids, and photosensitizers.
US9610250B2	Nanolipogel vehicles for controlled delivery of different pharmaceutical agents	Tarek M. Fahmy, Eric STERN, Richard A. Flavell, Jason Park, Alyssa Siefert, Stephen H. Wrzesinski	Discusses nanolipogel vehicles for the controlled delivery of various pharmaceutical agents.
US20080044484A1	Use of polymeric nanoparticles for vaccine delivery	Boris Minev	Describes the use of polymeric nanoparticles to deliver vaccines.
US20040038406A1	Nanoparticle delivery systems and methods of use thereof	Gretchen Unger, Beverly Lundell	Covers various nanoparticle delivery systems and their methods of use.

## Challenges and Limitations of Cancer Immunotherapy

Cancer immunotherapy has revolutionized cancer treatment by providing new hope in areas where conventional medicines have failed. However, several challenges and limitations still impede its widespread success, as shown in Table 4. One major challenge is the heterogeneity of tumors. Cancer is not a singular disease but a collection of disorders with diverse genetic and molecular profiles, complicating the development of universal immunotherapeutic strategies [255]. Additionally, the tumor microenvironment (TME) often has immunosuppressive characteristics that hinder the efficacy of immunotherapies. Elements within the TME, such as regulatory T cells (Tregs), myeloid-derived suppressor cells (MDSCs), and certain cytokines, can inhibit the activity of effector immune cells, reducing overall treatment effectiveness [256, 257].

The frequency of immune-related adverse events (irAEs) is another major problem. Even though immunotherapies such as checkpoint inhibitors are very new, they can cause serious autoimmune reactions because they cause the immune system to attack healthy tissues. These irAEs range from mild skin rashes to severe, life-threatening conditions affecting various organs, necessitating careful management and sometimes the discontinuation of therapy [258]. Furthermore, the development of resistance to immunotherapy poses a substantial hurdle. Some patients initially respond well to treatments but eventually develop resistance, leading to disease progression. This resistance can be intrinsic, where tumors do not respond from the outset, or acquired, where tumors initially respond but later relapse. The mechanisms underlying resistance are complex and include genetic mutations, alterations in antigen presentation, and adaptive immune resistance mechanisms [259, 260].

The cost and accessibility of immunotherapies are also significant concerns. These treatments are often expensive, limiting their availability to a broader population, especially in low- and middle-income countries. The complex manufacturing processes and the need for highly specialized administration further exacerbate these issues, creating disparities in treatment access [261, 262]. Therefore, while cancer immunotherapy holds tremendous promise, addressing the challenges of tumor heterogeneity, the immunosuppressive tumor microenvironment, immune-related adverse events, resistance mechanisms, and cost barriers are crucial for its future success and broader applicability.

## Conclusion and Future Perspectives

Cancer immunotherapy has become a revolutionary method for treating cancer, increasing patient survival rates and quality of life. The advent of immune checkpoint inhibitors, CAR-T-cell therapies, cancer vaccines, cytokine therapies, and oncolytic viruses has significantly broadened the therapeutic arsenal against various malignancies. Recent advances in delivery modalities have further enhanced the precision and efficacy of these therapies, allowing for more targeted and personalized treatment strategies. However, the journey of cancer immunotherapy is fraught with challenges. Issues such as immune-related adverse events, high costs, and variable patient responses underscore the need for a deeper understanding of the underlying mechanisms and the development of predictive biomarkers. Additionally, the complexity of the TME and the potential for immune escape mechanisms necessitate innovative approaches to overcome these hurdles.

Future research should focus on elucidating the molecular and cellular interactions within the tumor microenvironment, developing combination therapies to increase treatment efficacy and reduce resistance, and identifying novel biomarkers for better patient stratification. Advances in bioinformatics and systems biology will be crucial in unraveling the complexities of immune responses and tailoring treatments to individual patient profiles. Moreover, expanding the accessibility and affordability of cancer immunotherapies remains a critical goal. Collaborative efforts among academia, industry, and regulatory bodies are essential to streamline the development and approval processes, ensuring that these life-saving treatments reach a broader patient population.

In conclusion, while significant strides have been made in the field of cancer immunotherapy, the journey is far from over. By addressing the existing challenges and exploring new avenues, we can pave the way for more effective, safe, and accessible immunotherapies. The future of cancer treatment lies in the continued innovation and integration of immunotherapy into comprehensive cancer care, ultimately bringing us closer to a world where cancer is a manageable and curable disease.

## Data Availability

No datasets were generated or analysed during the current study.

## Abbreviations

APCs: Antigen-Presenting Cells
CAR-T: Chimeric Antigen Receptor T-cell
CTLA-4: Cytotoxic T-lymphocyte antigen 4
FDA: Food and Drug Administration
HPV: Human papillomavirus
IL: Interleukin
PD-1: Programmed death-1
PD-L1: Programmed death-ligand 1
QbD: Quality by Design
T cells: T lymphocytes
X-rays: X-radiation
HCV: Hepatitis C Virus
PAP: Prostatic acid phosphatase
GM-CSF: Granulocyte-Macrophage Colony-Stimulating Factor
IFNs: Interferons
NK: Natural killer (cells)
IL-2: Interleukin-2
IFN-α: Interferon-alpha
HSVs: Herpes simplex viruses
EPR: Enhanced permeability and retention
EpCAM: Epithelial cell adhesion molecule
HepG2: Human liver cancer cell line
MDSCs: Myeloid-derived suppressor cells
ICD: Immunogenic cell death
MHCs: Major histocompatibility complexes
CTLs: Cytotoxic T lymphocytes
EVs: Extracellular vesicles
TILs: Tumor-infiltrating lymphocytes
PNP: Polymer-Nanoparticle
CSPG4. CAR: Chondroitin sulfate proteoglycan 4 chimeric antigen receptor
MOFs: Metal-Organic Frameworks
